# Copy number variations in atypical fibroxanthomas and pleomorphic dermal sarcomas

**DOI:** 10.18632/oncotarget.22691

**Published:** 2017-11-25

**Authors:** Doris Helbig, Alexander Quaas, Cornelia Mauch, Sabine Merkelbach-Bruse, Reinhard Büttner, Michael Emberger, Marion Wobser, Vanessa Rüsseler, Katharina Pütz, Elke Binot, Jan Rehker, Jan Budczies, Michaela Angelika Ihle

**Affiliations:** ^1^ Department of Dermatology, University Hospital Cologne, Cologne, Germany; ^2^ Institute of Pathology, University Hospital Cologne, Cologne, Germany; ^3^ Institute of Pathology, Salzburg, Austria; ^4^ Department of Dermatology, University Hospital Würzburg, Würzburg, Germany; ^5^ Institute of Pathology, Charité University Hospital, Berlin, Germany

**Keywords:** atypical fibroxanthoma, BRAF, copy number variation, pleomorphic dermal sarcoma

## Abstract

Atypical fibroxanthomas (AFX) and pleomorphic dermal sarcomas (PDS) are frequent cutaneous sarcomas typically arising on sun-exposed skin in elderly patients. In contrast to AFX, which generally do not recur after complete excision, PDS locally recur in up to 50% and metastasize in up to 20%.

We recently detected characteristic UV-induced *TP53* mutations as potential driver mutation in almost all PDS investigated as well as activating *PIK3CA* and *RAS* gene mutations in around one third of our tumors representing targets for personalized treatments in patients with unresectable or metastasized PDS.

In the present study, we identified amplifications and deletions in a small part of the PDS (6 of 27 cases) but not in AFX suggesting that copy number variations (CNV) might not be an initial event in tumor development but rather important during tumor progression. In addition to *BRAF, KNSTRN, IDH1* and *PDGFRA* amplification, CNV analyses revealed deletions in the *CDKN2A*, *KIT* and *PDGFRA* genes. In cases where an appropriate FISH assay was established, the CNV results could be verified by FISH analysis.

Amplification of *BRAF, KIT* or *PDGFRA* and/or losses of *CDKN2A* might represent bad prognostic markers, although larger studies are needed to clarify their association with prognosis or progression in PDS.

## INTRODUCTION

Atypical fibroxanthomas (AFX) and pleomorphic dermal sarcomas (PDS) represent frequent subtypes of cutaneous sarcomas typically arising on sun-exposed skin of the head and neck in elderly patients. AFX generally do not recur after complete excision whereas PDS have a high potential for local recurrence, metastasis and disease-specific death in case of unresectable tumors [1, 2].

Using next generation sequencing (NGS)-based mutation analyses, we recently detected characteristic UV-induced *TP53* mutations as potential driver mutation in almost all our PDS investigated. Besides, we detected *CCND1*/*CDK4* alterations, *PIK3CA* and different *RAS* gene mutations as well as an *ALK* translocation as additional underlying genetic alterations representing targets for personalized treatments in patients with unresectable or metastasized PDS [2].

In addition to somatic mutations, copy number variations (CNV) by gain of specific chromosomal segments containing relevant oncogenes or loss of chromosomal segments harboring critical tumor suppressor genes have been shown to be highly characteristic of other UV-induced skin tumors such as malignant melanoma [3], cutaneous squamous cell carcinoma (SCC) [4] and basal cell carcinoma (BCC) [5]. The aim of the present study was to analyze CNV in a large sample cohort of AFX and PDS to get further insights into their evolutionary process as well as to detect additional diagnostic or therapeutic target structures.

## RESULTS

### Detection of mutations by next generation sequencing

The results of all NGS analyses are summarized in Table 1 and are partly published by Helbig et al. [2].

**Table 1:** Results of the NGS, CNV and FISH analyses

Case no.	NGS result	CNV gain	CNV loss	FISH result
A1	*TP53*: c.658T>C p.Y220H	none	none	
A2	*KRAS*: c.35G>A p.G12D	none	none	
A5^1^	*BRAF*: c.1799T>A p.V600E; *PIK3CA*: c.1624G>A p.E542K; *TP53*: c.672+1G>C; TP53: c.818G>A p.R273H	none	none	
P1	*KNSTRN*: c.28G>A p.D10N; *TP53*: c.585_586delinsTT p.R196^*^; *TP53*: c.748_749CC>TT p.P250F	none	none	
P2	*TP53*: c.[734G>A];[741C>T] p.[G245D];R248W]	none	none	
P3	*PIK3CA*: c.1600A>G p.I534V; *TP53*: c.464_465CC>TT p.T155I; *TP53*: c.853G>A p.E285K; *TP53*: c.783-2A>T	none	none	
P4	*OXA1L*: c.169C>T p.L57F; TP53: c.773A>C p.E258A; *TP53*: c.808T>A p.F270I	none	none	
P5	*TP53*: c.574C>T p.Q192^*^; *TP53*: c.742C>T p.R248W	*BRAF* (CN=4.5, p=1.4e-23)	none	*BRAF* ratio 4.3
P6	*TP53*: c.949C>T p.Q317^*^	none	none	*BRAF* ratio 1.2; *CDK4* ratio 1.9; *KIT* ratio 1.8; *PDGFRA* ratio 1.4
P7	*TP53*: c.530C>T p.P177L; *TP53*: c.742C>T p.R248W	none	none	
P8	wildtype	none	none	*BRAF* ratio 1.0; *CDK4* ratio 0.85; *KIT* ratio 0.7; *PDGFRA* ratio 1.0
P9	*RAC1*: c.85C>T p.P29S; *TP53*: c.380C>T p.S127F; *TP53*: c.585C>T p.R196^*^	*KNSTRN* (CN=3.7, p=2.7e-14)	none	
P10	*KRAS*: c.38G>A p.G13D; *TP53*: c.406C>T p.Q136^*^; *TP53*: c.949C>T p.Q317^*^	none	none	
P11	*KNSTRN*: c.13G>A p.E5K; *PIK3CA*: c.1633G>A p.E545K; *TP53*: c.644G>C p.S215T; *TP53*: c.730G>A p.G244S; *TP53*: c.833C>T p.P278L	none	none	*BRAF* ratio 1.2; *CDK4* ratio 1.0; *KIT* ratio 1.9; *PDGFRA* ratio 1.3
P13	*HRAS*: c.38G>A p.G13D; *TP53*: c.747G>T p.R249S	none	none	
P14	*TP53*: c.856G>A p.E286K; *TP53*: c.841G>A p.D281N	none	none	
P15	*KNSTRN*: c.13G>A p.E5K; *TP53*: c.581T>G p.L194R	none	*CDKN2A* (CN=0.5, p=0.037)	
P16	*TP53*: c.528C>G p.C176W; *TP53*: c.580C>T p.L194F	none	none	
P18	*NRAS*: c.181C>A p.Q61K; *PIK3CA*: c.3145G>A p.G1049S; *TP53*: c.422G>T p.C141F; *TP53*: c.655C>T p.P219S; *TP53*: c.743_744GG>AA p.R248Q	*IDH1* (CN=2.9, p=7.7e-06)	*PDGFRA* (CN=1, p=0.0031); *KIT* (CN=0.8, p=3.5e-05)	*KIT* ratio 0.6; *PDGFRA* NA
P19	*RAC1*: c.85C>T p.P29S; *TP53*: c.535C>T p.H179Y; *TP53*: c.586C>T p.R196^*^	none	none	
P20	*TP53*: c.859G>T p.E287^*^	none	none	
P21	*PIK3CA*: c.1624G>A p.E542K; *TP53*: c.584_585TC>GT p.I195S; *TP53*: c.716A>G p.N239S	none	none	
P22^1^	*PIK3CA*: c.1624G>A p.E542K; *TP53*: c.672+1G>C; *TP53*: c.818G>A p.R273H	none	none	
P23	*IDH1*: c.379C>T p.P127S; *TP53*: c.637C>T p.R213^*^	none	none	
P24	*TP53*: c.585_586CC>TT p.R196^*^; *TP53*: c.712T>A p.C238S	none	*PDGFRA* (CN=1.4, p=0.0034)	*PDGFRA* ratio 0.4
P25	*TP53*: c.490A>T p.K164^*^; *TP53*: c.586C>T p.R196^*^	PDGFRA (CN=4.2, p=7.1e-25)	none	PDGFRA ratio 3.8
P26^1^	*PIK3CA*: c.1624G>A p.E542K; *TP53*: c.672+1G>C; *TP53*: c.818G>A p.R273H	none	none	

^1^ 1 AFX (Case A5) and two recurring PDS samples (Case P22 and P26) came from the same patient at the age of 83, 86 and 87 years.

NA=Not applicable, Cases A3, A4 and P12, P17 were not analyzable due to insufficient tumor material.

### Copy number variations detected by ioncopy

**Figure 1:**
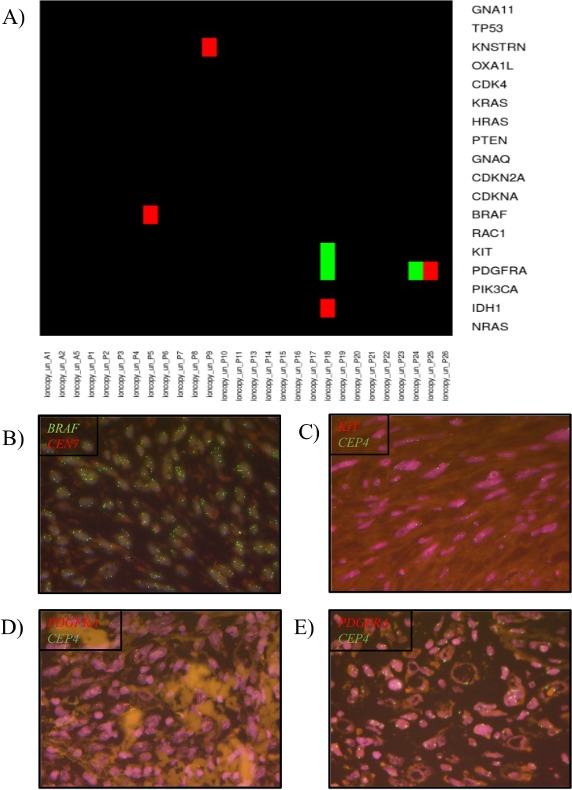
Copy number variation (CNV) in pleomorphic dermal sarcomas (PDS) **(A)** Heatmap diagram of the CNVs in a melanoma specific gene panel detected by the software tool Ioncopy. Red indicates an amplification and green a deletion. **(B)** Verification of *BRAF* amplification by FISH analysis. **(C)** Verification of *KIT* deletion by FISH analysis. **(D)** Verification of *PDGFRA* amplification by FISH analysis. **(E)** Verification of *PDGFRA* deletion by FISH analysis.

One PDS harbored a *BRAF* amplification (CN=4.5, p=1.4e-23, ndetected=3/5, mean_CN=4.1; see materials and methods for details, case P5) (Figure 1). In addition, a *KNSTRN* amplification (CN=3.7, p=2.7e-14, ndetected=2/3, mean_CN=3.2, case P9), an *IDH1* amplification (CN=2.9, p=7.7e-06, ndetected=1/2, mean_CN=2.5, case P18) and a *PDGFRA* amplification PDGFRA (CN=4.2, p=7.1e-25, ndetected=4/8, mean_CN=3.6, case P25) was detected.

Deletions could be detected in 2 tumors (Figure 1). Simultaneous *PDGFRA* (CN=1, p=0.0031, ndetected=4/8, mean_CN=1.5) and *KIT* (CN=0.8, p=3.5e-05, ndetected=7/14, mean_CN=1.5) deletions were detected in case P18. Single gene copy number deletions of *PDGFRA* (CN=1.4, p=0.0034, ndetected=5/8, mean_CN=1.4, case P24) was identified in one PDS.

In all other tumors including all AFX and the recurring case (AFX= case A5, PDS= case P22 and P26) no CNV could be detected. For details see Table 1. In the whole cohort no simultaneous mutation and CNV was detected in the same gene. *BRAF* CNV gain was detected with *TP53* mutations, *KNSTRN* CNV gain with *RAC1* and *TP53* mutations, *IDH1* with *NRAS*, *PIK3CA* and *TP53* mutations and simultaneous *PDGFRA* and *KIT* CNV loss, *PDGFRA* with *TP53* mutations. Single CNV loss in *PDGFRA* was accompanied with *TP53* mutations.

### Verification of the copy number variations by fluorescence *in-situ* hybridization

To verify our Ioncopy results we compared the bioinformatically obtained CNV results with those determined by the current gold standard fluorescent *in-situ* hybridization (FISH). All amplified or deleted samples were analyzed by FISH if an appropriate FISH assay was established in our institute. To exclude false negative samples FISH was performed exemplarily in three cases.

The *BRAF* amplification in the PDS case P5 could be confirmed by FISH analysis (Figure 1B). The case showed a ratio of 4.3 and a gene copy number of ≥ 6.0. The PDS with PDGFRA amplification (case P25) was also positive by FISH analysis showing a ratio of 3.8.

Case P18, with a simultaneous *PDGFRA* and *KIT* deletion, showed for *KIT* a ratio of 0.6 in the FISH analysis confirming the deletion detected by Ioncopy (Figure 1C). The *PDGFRA* FISH could not be analyzed due to low signal intensity even after repetition.

The CNV loss of *PDGFRA* detected by Ioncopy in case P24 could be confirmed by FISH analysis showing a ratio of 0.4.

The single copy number variations detected in *KNSTRN* (case P9), *IDH1* (case P18) were not verified due to the lack of an appropriate FISH assay.

**Figure 2:**
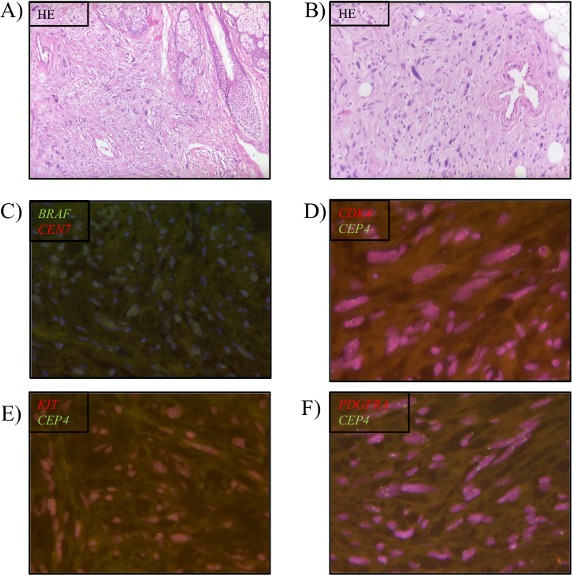
Verification of negative case P6 Negative results by Ioncopy were verified by FISH analysis if an appropriate assay was available. **(A, B)** HE staining, **(C)** *BRAF* FISH, **(D)** *CDK4* FISH, **(E)** *KIT* FISH, and **(F)** *PDGFRA* FISH.

Four bioinformatically negative cases were also analyzed by FISH for *BRAF*, *CDK4*, *KIT* and *PDGFRA* copy number variations to exclude false negative samples. The negative results could be confirmed in FISH assays in all samples. Figure 2 shows exemplarily the negative FISH results of case P6. This case showed a ratio of 1.2 for *BRAF*, one of 1.9 for *CDK4*, one of 1.8 for *KIT* and one of 1.4 for *PDGFRA* FISH analysis.

## DISCUSSION

In the present study, we used amplicon sequencing data generated on a MiSeq Benchtop sequencer to perform CNV analysis by the software tool Ioncopy. As described by Budczies et al. the detection of amplifications is possible with one amplicon covering the region of interest and without corresponding normal tissue [6]. However, confidences increase when the region of interest is covered by at least two amplicons. Furthermore, it is essential that the raw data is carefully inspected before analysis to exclude bad quality cases. A minimum coverage of 100x for all amplicons is recommended but the reliability increases with the coverage. Ioncopy is a good tool to analyze the CNVs without normal control tissue with a minimum of 20 samples as shown in the present study. All CNVs detected by Ioncopy could be verified by FISH analysis if an appropriate FISH assay was established.

Characteristic CNV have been identified in other UV-induced skin tumors such as malignant melanomas [3], cutaneous SCC [4] and BCC [5]. In only one previous study comparative genomic hybridization (CGH) revealed many shared CNVs in AFX and PDS (most frequently deletions on chromosomes 9p and 13q). Overall, the number of CNVs detected in PDS was higher than in AFX (8.8 ± 1.1 versus 3.3 ± 0.7) [7]. CNVs on single gene level have not been analyzed yet in AFX and PDS.

In the present study, we identified amplifications and deletions in a small part of the PDS suggesting that CNVs might not be an initial event in tumor development but rather are important during tumor progression. The analyzed AFX did not show any CNVs.

The *BRAF* amplification detected in one PDS may play an essential role in this tumor. The *BRAF* gene encodes the serine/threonine-protein kinase BRAF, which regulates normal cell growth and proliferation. It is frequently mutated in cancer with highest mutation rates in hairy cell leukemia, malignant melanoma, and papillary thyroid cancer [8–10]. Overall, the most frequent *BRAF* mutation is V600E which leads to a constitutive activation of the BRAF kinase and its downstream signaling through the mitogen-activated protein kinase (MAPK) pathway [8, 11]. Two BRAF inhibitors (vemurafenib and dabrafenib) and one MEK inhibitor (trametinib) are recently approved for the treatment of metastatic *BRAF* V600E mutated melanoma [12–18]. Following this, a clinically relevant antitumor activity of these BRAF inhibitors could also be demonstrated in patients with advanced V600E-mutant non–small-cell lung cancer, thyroid cancer, and hairy cell leukemia [19, 20]. Despite a prolonged disease free and patient’s overall survival under those BRAF inhibitors, the combination with a MEK inhibitor is useful to delay acquired drug resistances [21, 22]. *BRAF* amplifications have been described in various frequencies in melanomas (5.6-66%) and seemed to be associated with decreased progression free survival (PFS), independent of the underlying *BRAF* mutation [23]. More frequently, *BRAF* amplifications have been identified as a key regulator for treatment resistance to *BRAF* and *MEK* inhibitors, either alone or in combination [23–27]. In colorectal carcinoma, *BRAF* amplification induced resistance to RAF/EGFR or RAF/MEK combinations through sustained MAPK pathway activity. Interestingly, an ERK inhibitor was able to overcome this resistance [28]. As a *BRAF* amplification is associated with decreased response to *BRAF* and or *MEK* inhibition and poor clinical outcome, this specific genetic alteration may serve as a predictive biomarker. Furthermore, treatment regimens and combinations should be adapted to this specific molecular alteration to prevent resistance.

In addition to this *BRAF* amplification, our CNV analyses revealed amplifications in *PDGFRA* as well as deletions in *KIT* and *PDGFRA*. *KIT* (CD117) and *PDGFRA* (both localized on chromosome 4q) encode for two homologous receptor tyrosine kinases. Oncogenic mutations in *KIT* occur in 75-80% of gastrointestinal stromal tumors (GIST) and in 20-25% in *PDGFRA* [29]. GIST with a mutation in either *KIT* or *PDGFRA* can be treated with selective small inhibitors such as Imatinib, Sunitinib or Regorafenib [30–32]. *KIT* copy number variations are described in melanoma, especially in acral (27.3%) and mucosal (26.3%) melanoma [33–35], in glioblastoma multiforme [36] and in intimal sarcoma [37]. They are detected together with *BRAF* V600E, *NRAS* or *KIT* mutations or even alone [38]. In the study of Minor et al., a patient with a *KIT* amplification and *NRAS* mutation, as shown in the present study, showed progressive disease under Sunitinib treatment [38]. In the whole cohort, only one of the six detected patients with *KIT* amplification had a partial response. In the study of Hodi et al., none of the patients with *KIT* wildtype amplification responded to Imatinib treatment [39]. In a multicenter phase II study with Nilotinib, a novel phenylaminopyrimidine derivate with potent activity against KIT, *KIT* amplification alone was detected in 35.7% of melanoma and combined with *KIT* mutations in 4.8%. Seven patients responded to Nilotinib treatment and one out of these seven harbored a *KIT* amplification only [40]. Therefore, the presence of not only a *KIT* mutation but also a *KIT* amplification seems to be a poor prognostic marker [41, 42].

*PDGFRA* amplifications are also described as a bad prognostic marker in adult *IDH1* mutated high-grade glioma [43] and adult anaplastic astrocytoma [44]. *In vitro* studies suggest Dasatinib as a good therapeutic option for *PDGFRA* amplified pediatric high-grade glioma [45].

Beside this, we could detect CNV gain in *KNSTRN* and *IDH1*. *KNSTRN* (kinetochore-localized astrin-binding protein) encodes a kinetochore-associated protein that regulates anaphase onset and chromosomal segregation during mitosis. Point mutations in *KNSTRN* are described in cutaneous squamous cell carcinoma. Here, the hotspot mutation in codon 24 leads to a disrupted sister chromatin cohesion, an increased tumor aneuploidy and tumor growth *in vivo* [40]. To our knowledge, *KNSTRN* CNV gain not yet described in the literature so that the clinical significance of this aberration is not known.

*IDH1* (Isocitrate dehydrogenase 1) is located on chromosome 2q33.3 and encodes for a protein that catalyzes the reversible oxidative decarboxylation of isocitrate to alpha-ketoglutarate (αKG) while reducing NADP+ to NADPH. Mutations in the *IDH1* gene (mainly in codon 132) are described in solid tumors as well as in haematologic malignancies such as glioma, acute myeloid leukemia and chondrosarcoma [46]. In low grade gliomas *IDH1* mutations are the earliest genetic alteration whereas higher grade gliomas are associated with *IDH1* copy number variations [47]. Therefore, *IDH1* copy number variation seems to be a worse prognostic biomarker.

Based on these results and our findings, an amplification of *BRAF* and/or losses of *PDGFRA* as well as *KIT* could represent markers for worse clinical outcome, although larger studies are needed to clarify their association with prognosis or progression in PDS.

As we already suggested earlier, a subset of patients (showing gene mutations in *PIK3CA*, *HRAS*, *KRAS*, *NRAS* and *BRAF* or *ALK* translocation) could benefit from a systemic treatment with a *PI3K, MEK, BRAF* or *ALK* inhibitor in the case of unresectable or metastasized tumor stage [2].

## MATERIALS AND METHODS

### Patient characteristics and tumor material

3 AFX and 24 PDS, initially diagnosed in different dermatopathology centers (Department of Dermatology, University Hospital Cologne, Germany; Institute of Pathology Weger Emberger OG, Salzburg, Austria; Department of Dermatology, University Hospital Würzburg, Germany) were included. Cases A3, A4 and P12, P17 were not analyzable due to insufficient tumor material. One patient developed an AFX (case A5) which recurred twice as PDS (case P22 and P26) 3 and 4 years after the initial diagnosis. All tumors were selected and reevaluated by D.H. and A.Q. according to our definitions and part of the cohort has been already described before [2]. For all patient and tumor details see Table 2.

**Table 2:** Patient and tumor characteristics

	PDS (n= 26^1^)	AFX (n=5^1^)
**Male**	23	5
**Female**	3	
**Age (Years)**		
**-** **range**	58-94	63-83
**-** **median**	80.5	76
**-** **mean**	79	76
**Tumor localization**		
**-** **Capillitium/Face**	25	4
**-** **Shoulder**	1	
**-** **Not known**		1

^1^ 1 AFX and 2 recurring PDS samples came from the same patient at the age of 83, 86 and 87 years.

### Next-generation-sequencing (NGS)

NGS analysis has been described earlier [2].

### Copy number variations (CNV)

CNVs were detected with the freely available software tool Ioncopy [6]. This method allows the detection of CNVs based on amplicon sequencing data. The advantage of this method is that no normal controls are needed and that somatic mutations and CNVs (amplifications and deletions) can be detected from the same raw data. Coverage files were loaded into Ioncopy to detect CNVs. They were generated from the same BAM files as for mutational analysis generated by a MiSeq benchtop sequencer (Illumina, [48]). Copy numbers were estimated after sample and amplicon normalization. Significance of CNVs was assessed separately for each amplicon including only amplicons with mean coverage ≥ 100 in the analysis. Similar to Budczies et al. an amplification was called, when detected by a single amplicon and a significance (p<0.05) after Bonferroni correction for the total number of samples and amplicons [6]. A deletion was called, when detected by at least four amplicons (p<0.05 without multiple testing correction for each of them). For each gene called, CN and p-value of the most significant amplicon, the number of significant amplicons compared to the total number of the amplicons interrogating the gene (ndetected) and average CN over all amplicons (mean_CV) were reported.

Quality criteria were a mean coverage ≥ 100 and a significance of copy number variation of p = 0.05. For each CNV the copy number (CN) with significance (p=0.05) is described. Ndetected gives the number of amplicons with a significant amplification compared to the total number of the amplicons covering a gene. Mean_CV describes the mean of copy numbers of the total amplicons of one gene.

### Fluorescence *in-situ* hybridization (FISH)

Four μm thick sections from formalin-fixed and paraffin embedded (FFPE) tissue blocks were mounted on sialinized slides for FISH analysis.

Briefly, pretreatments and washes were performed on the half-automated VP2000 processor system (Abbott Molecular, Wiesbaden, Germany). The slides were then denatured for five minutes at 75°C and incubated with the appropriate probes at 37°C overnight: ZytoLight^®^ Spec *BRAF*/*CEN7* dual color probe (purchased from ZytoVision GmbH, Bremerhaven, Germany) for *BRAF* copy number variation, SureFISH 4q12 *KIT* 155 kb, orange-red probe combined with SureFISH Chr4 *CEP* 613 kb, green probe (both purchased from Agilent Technologies, Cedar Creek, USA) for *KIT* copy number variations and in-house made *PDGFRA* or *CDK4* probes (Bacterial artificial chromosome clone: RP11-XY labeled with Spectrum Orange or bacterial artificial chromosome clone: RP11-571M6 labeled with Spectrum Orange both combined with a centromere specific Spectrum Green probe) for *PDGFRA* or *CDK4* CNV`s.

Unbound probes were washed away with 2x post-hybridization SSC buffer at 72°C and nuclei were counterstained with DAPI (Sigma-Aldrich GmbH, Taufkirchen, Germany). Fluorescent signals of twenty non-overlapping contiguous tumor cells were analyzed in random areas with a DM5500B fluorescent microscope (Leica, Wetzlar, Germany). The ratio of *BRAF*/*CEN7*, *KIT*/*CEN4*, *PDGFRA*/*CEN4* or *CDK4*/*CEN12* signals and the average gene copy number were estimated for each sample. Criteria for an amplification were either a ratio ≥ 2.0 or an average gene copy number per cell of ≥ 6.0, criteria for a deletion was a ratio < 0.8.
